# A Rare Case of Mid-clavicular Ewing Sarcoma Treated With Total Claviculectomy: A Case Report and Literature Review

**DOI:** 10.7759/cureus.78979

**Published:** 2025-02-14

**Authors:** Khaled K AlAbbasi, Mustafa AlRawi, Osama Alshaya

**Affiliations:** 1 Orthopedic Surgery Department - Upper Limb Section, King Fahad Medical City, Riyadh, SAU; 2 Orthopedic Surgery Department - Orthopedic Oncology and Arthroplasty Sections, King Fahad Medical City, Riyadh, SAU

**Keywords:** clavicle ewing sarcoma, clavicle tumor, claviculectomy, ewing sarcoma (es), upper-limb malignancy

## Abstract

Ewing sarcoma of the clavicle is an extremely rare tumor. The currently available evidence is limited to a small number of case reports. We present a case of a 13-year-old female patient referred to our tertiary care center with a progressively growing lump over the mid-clavicular, mystically diagnosed as a benign lipoma in a rural hospital. Radiological investigations and histopathological examination confirmed the diagnosis of metastatic Ewing sarcoma of the mid-clavicle. We outlined the management plan done for this patient followed by a description of the surgical technique of the total claviculectomy performed. Furthermore, we conducted a literature review on the available evidence on this topic and the functional outcomes of partial and total claviculectomy with or without reconstruction.

## Introduction

Bones of the body are categorized into long bones, such as the femur, tibia, and humerus, and flat bones such as the pelvis, scapula, and ribs. Each of these two major categories has characteristic histological, embryological, and developmental features that predispose them to a specific group of primary bone tumors [1]. The clavicle is special in sharing some characteristics of both long and flat bones. Developmentally, like flat bones, the clavicle diaphysis ossifies by intermembranous ossification, while the two ends ossify by endochondral ossification, like long bones [1,2]. Anatomically, the clavicle is a long bone but is special in lacking a definitive medullary canal and in being the only long bone in the horizontal axis of the body. Furthermore, studies have shown a higher tendency of tumors and tumor-like lesions at the acromial end of the clavicle, followed by the medial sternal end, with rare occurrence in the mid clavicle, a distribution that resembles long bones [1,3]. These mixed characteristics result in the lack of predisposition to specific groups of bone tumors.

Tumors of the clavicle are rare, accounting for less than 1% of all skeletal tumors with a higher prevalence of malignant tumors compared to other parts of the skeletal system [1,2]. This rarity is reflected by the significantly limited number of studies reporting on tumors of the clavicle, mostly being case reports and a limited number of case series [1-3]. These facts, in addition to the lack of predisposition to specific types of bone tumors and the low level of suspicion, result in the lack of experience in clavicular tumor presentation, diagnosis, and management. In this study, we are reporting a case of Ewing sarcoma of the clavicle in a young female teenager with an unusual presentation and an extremely rare site. Furthermore, we outline the surgical technique of total claviculectomy performed for this patient, followed by a literature review on Ewing sarcomas of the clavicle and the reported functional outcomes after claviculectomy with and without reconstruction.

## Case presentation

**Figure 1:**
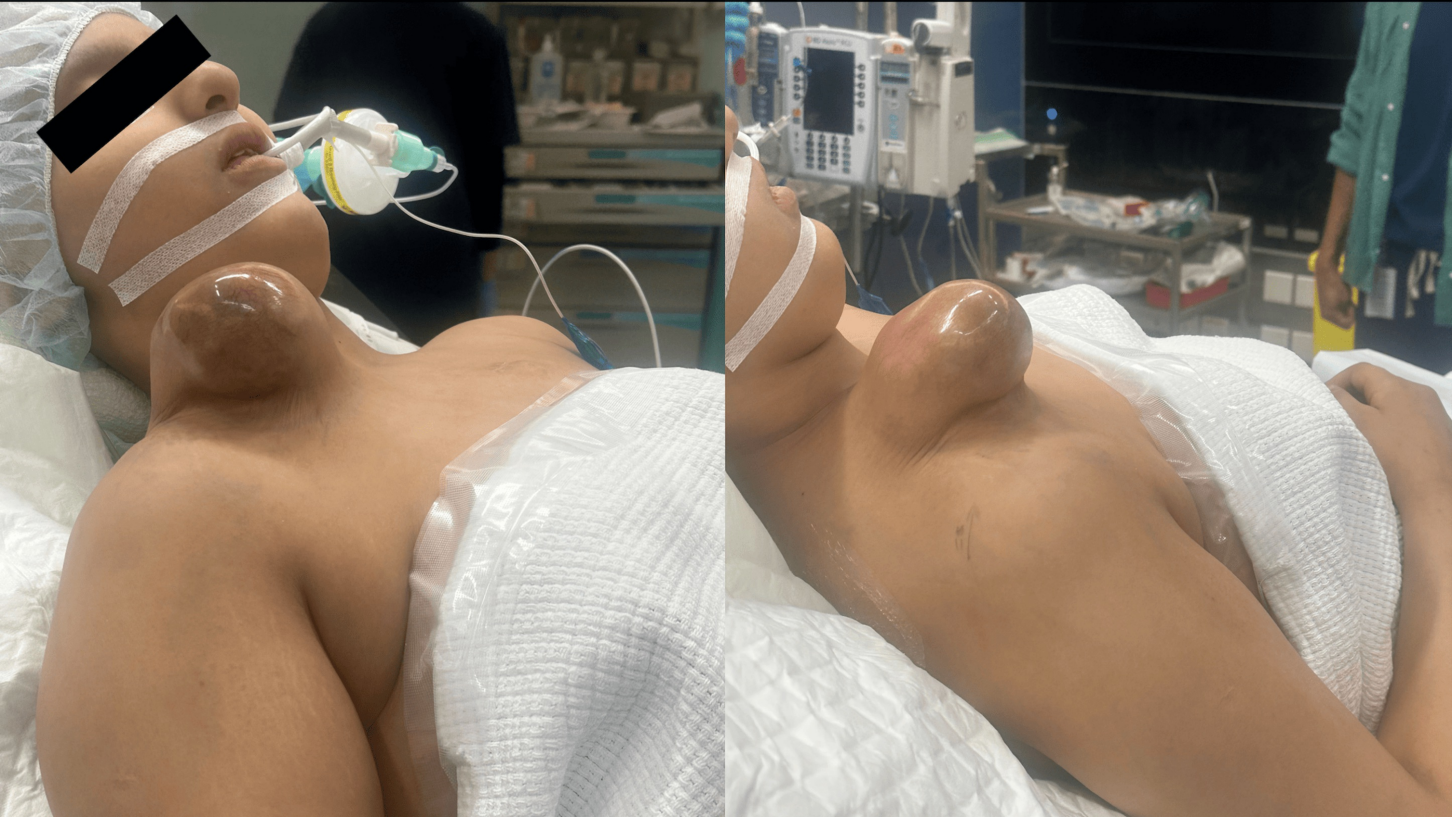
Clinical appearance of the tumor The picture was taken by the author after securing consent from the patient for taking images for medical and research reasons.

A 13-year-old female patient was referred to the orthopedic oncology outpatient clinic complaining of a progressively enlarging limp over the right clavicular area. This lump was initially diagnosed in a rural hospital for being a benign lipoma, but the progressive enlargement of the lump mandated transferring the patient to a tertiary care center (Figure 1). 

**Figure 2:**
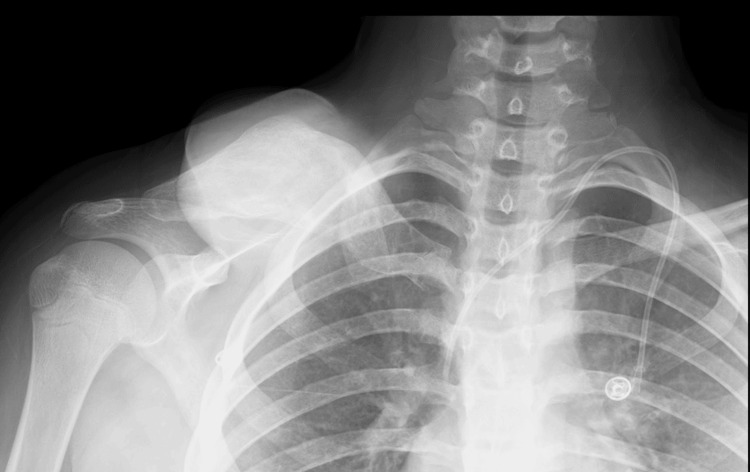
Chest X-ray of the patient before chemotherapy The images were taken from the patient's electronic records after securing consent from the patient for the publication, reproduction, broadcast, and other use of imaging and photos.

**Figure 3:**
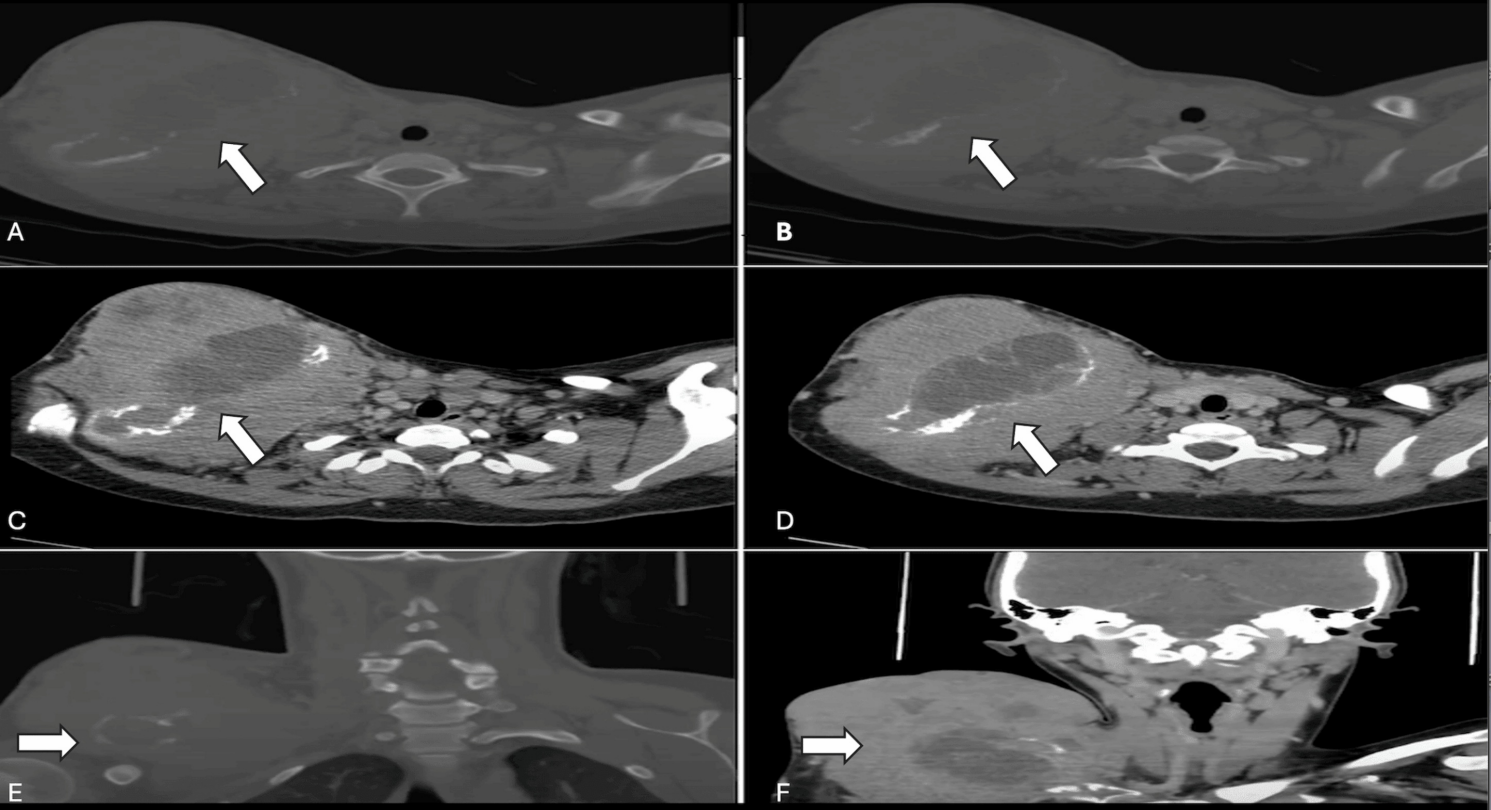
CT scan of the right clavicle before chemotherapy The axial bone view cuts (A and B) show the extensive destruction of the clavicle by the lytic lesion (white arrow). The axial soft tissue view cuts (C and D) show the soft tissue component of the lytic lesion along with the mass effect on the nearby vessels and structures (white arrow). The coronal views (E and F) show a large destructive lytic lesion emerging from the mid-clavicular lesion (white arrow). The images were taken from the patient's electronic records after securing consent from the patient for the publication, reproduction, broadcast, and other use of imaging and photos.

Upon presenting to our clinic, an oncology workup was initiated. The chest, abdomen, and pelvis CT scan reported the presence of a large, destructive, right clavicular heterogeneous soft tissue mass measuring 14.1x15.5x15 cm in maximum transverse, anteroposterior, and craniocaudal dimensions, respectively, with the presence of multiple prominent and large mesenteric lymph nodes. A head and neck CT scan revealed multilevel enlarged cervical lymph nodes with a mass effect of the tumor on the neck and abutment on the subclavian vein (Figures 2, 3).

Apart from the enlarged mesenteric and cervical lymph nodes, a positron emission tomography (PET)-computed tomography (CT) scan confirmed no other sites of metastasis. MRI was not done due to the patient's refusal. The diagnosis of metastatic Ewing sarcoma was confirmed by an ultrasound needle biopsy. A tumor board meeting was held to personalize a management plan for the patient. The patient underwent seven cycles of chemotherapy, alternating between the VAC (vincristine, actinomycin-D, cyclophosphamide) and VC (vincristine, cyclophosphamide) regimens. Post-chemotherapy radiological imaging confirmed a significant shrinkage in the size of the tumor. Surgery was planned three weeks after the last chemotherapy cycle.

Surgical technique

Surgery was performed under general anesthesia. The patient was placed in a supine position with the head elevated at 30 degrees. A small sandbag was placed between the two shoulders posteriorly. The ipsilateral arm and the chest were draped lateral to the contralateral edge of the manubrium in a fashion that allowed free movement of the entire limb. The edges of the surgical field and the axilla were covered with an Ioban drape (3M, St. Paul, MN, USA).

The sternoclavicular and acromioclavicular joints were identified and marked. An elliptical skin incision was made between the two joints. The skin over the tumor was included in the en-bloc resection. The plastic surgery team was consulted preoperatively for possible skin grafting since excising the skin over the tumor was more important than any concern of skin closure at the end of the procedure. Skin flaps were raised on both edges of the elliptical incision to expose the ends of the clavicle and the fascia of the muscles around it. The “safe zone” of dissection was marked, which was identified as the area at least 3 cm far from the tumor edges circumferentially. To ensure a “margin-free” en-bloc resection, the muscles in the safe zone were detached directly off the bone while the muscles around the tumor were resected as en bloc with the tumor using cautery to maintain the homeostasis and blunt dissection to avoid injury of the nearby neurovascular structures. Claviculectomy was started laterally and then proceeded medially since the major neurovascular structures are found around the medial half of the clavicle, so if an injury happens to any of these structures, it will be easier and faster to complete the claviculectomy to expose these structures and repair them.

The resection was started by dissecting the trapezius muscle off the upper aspect of the clavicle and the deltoid off the anteroinferior aspect. The clavicle was mobilized laterally by incising the capsule around the acromioclavicular joint and the two coracoclavicular ligaments. Dissection was then progressed medially within the safe zone around the tumor. Medially, the sternocleidomastoid muscle was detached from the superior aspect of the clavicle and the pectoralis major muscle from the anteroinferior surface. Meticulous care was taken not to injure the external or internal jugular veins when dissecting the sternocleidomastoid muscle. Mobilization of the clavicle medially was done by dissecting the capsule around the sternoclavicular joint.

The clavicle was then elevated at both ends. This exposed the subclavius muscle on the posterior surface of the clavicle. The muscle was dissected meticulously to avoid injury to any of the major vessels and nerves running under the clavicle, especially in our case where the tumor was putting pressure on the subclavian vein as seen in the CT angiography. Special attention was applied to identify and ligate the vessels that feed the tumor and are usually found posterior to the clavicle since those can be a major source of uncontrolled bleeding. The vascular surgeon was informed before starting the procedure as a heads-up in case of an emergency.

**Figure 4:**
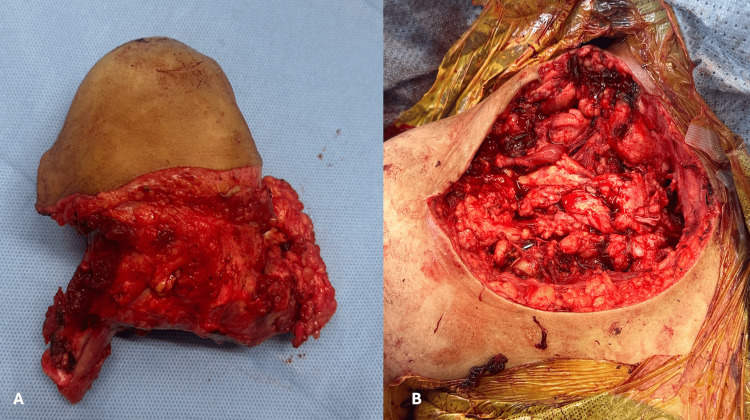
Gross pathology of the tumor after performing total claviculectomy with en-bloc resection of the tumor and the surrounding muscles with a 3 cm safety margin circumferentially (A). The supraclavicular region after performing total claviculectomy (B). The patient consented to the publication, reproduction, broadcast, and other use of imaging and photos.

After completing the claviculectomy, the clavicle with the tumor, the surrounding muscle, and overlying skin were taken to a side table to mark the tumor sides before sending it to pathology. Care was taken to ensure good homeostasis control. Irrigation of the wound was done using copious amounts of normal saline. To preserve the function of the upper limb, the deltoid was sutured to the trapezius muscles and the sternocleidomastoid to the pectoralis major. The skin was then closed in layers over a drain. Pressure dressing was finally applied (Figure 4). After performing total claviculectomy with en-bloc resection of the tumor and the surrounding muscles with a 3 cm safety margin circumferentially, histopathology reports confirmed the presence of small round cells consistent with Ewing sarcoma.

**Figure 5:**
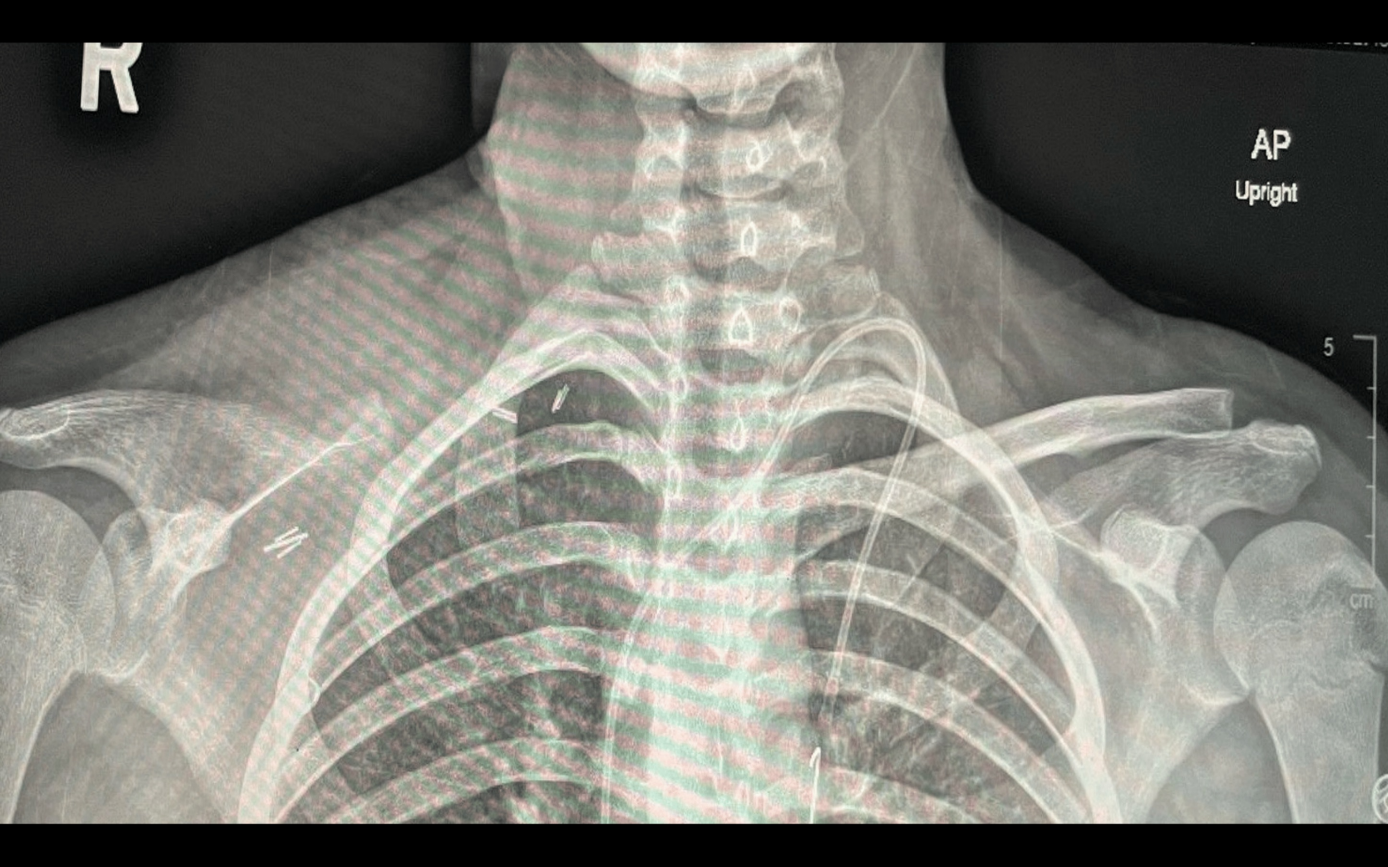
Postoperative X-ray The patient consented to the publication, reproduction, broadcast, and other use of imaging and photos.

Postoperatively, the patient was discharged home on day three after the removal of the drain. Skin sutures were removed in the third week. Minimal shoulder movements were allowed immediately after surgery. Physiotherapy was started at week 4 postoperatively, aiming for pain alleviation modalities and the increase of range of motion in the first phase, followed by strengthening and occupational therapy in the second phase. The patient continued to follow up with medical oncology for postoperative chemotherapy. Figure 5 shows a postoperative X-ray.

## Discussion

Ewing sarcoma is the second most common malignant osseous neoplasm in children and young adults [4]. It is of neuroectodermal origin and composed of small round cells with an increased nuclear-cytoplasmic ratio representing a family of small round blue cell tumors. Other tumors and Ewing sarcoma share morphological similarities with round-cell neoplasms with a common chromosomal translocation. Thus, the lesion category is termed the Ewing sarcoma family of tumors [5].

Ewing sarcomas account for about 5% of all soft tissue sarcomas, with a peak incidence ranging from 10 to 15 years of age. The most common anatomical sites include the pelvis, axial skeleton, and femur. Clavicular involvement is extremely rare and accounts for less than 1.4% to 1.6% of all Ewing sarcomas and 7.8% to 13.6 % of all clavicle tumors [1,6-10]. The most common involvement sites are the acromial end followed by the sternal end. While some limited numbered case reports showed no difference in the distribution of malignant tumors across the clavicle [2,9], larger studies indicate that involvement of the diaphysis of the clavicle is extremely rare [1,3,10]. No study reported the actual percentage of the distribution of Ewing sarcoma across the clavicle.

Typically, patients present with mild to moderate pain and swelling over the site of involvement. Patients may present with severe throbbing pain due to the involvement of the nearby supraclavicular nerve [2]. Fever is not uncommon, presenting in 15% to 20% of cases [11]. Nevertheless, the presence of a growing mass remains the most common presentation due to the superficial location of the clavicle. X-rays and CT scans usually reflect the aggressiveness of this tumor in the form of a moth-eaten lesion with a periosteal reaction resembling an onion peel pattern that reflects the new layers of bone formed. The MRI shows a heterogeneous mass due to the presence of necrosis or hemorrhage in the center of the mass [12]. PET-CT is usually recommended for local and systematic staging. The gold standard for diagnosing Ewing sarcoma in the clavicle is a histological examination after performing a biopsy of the lesion. It is advised to avoid needle biopsies and perform biopsies under fluoroscopic guidance to avoid injuring any of the nearby neurovascular structures [10,13].

Currently, multi-modal therapeutic strategies are used in the management of Ewing sarcoma, including chemotherapy before and after surgery, radical excision surgery, and radiation in some cases. Management is started using induction chemotherapy to decrease the size of the tumor followed by surgical excision within 10-12 weeks of the initiation of chemotherapy [5]. Radiation is usually given for cases with poor response to chemotherapy, presenting with a pathological fracture, or those presenting with a large soft tissue component [5,14]. 

The main goal of surgical management is to achieve local control by performing a radical excision of the tumor while ensuring free margins. Surgical management of clavicular tumors includes partial and complete claviculectomy without or with reconstruction using an allograft, a vascularized bone graft, or a plate-cement complex. Advocates of reconstructing the clavicle after partial or total calviculectomy emphasize the important role of the clavicle as a protective structure for the brachial plexus and great vessels present underneath and in ensuring proper function of the shoulder joint [5,15]. No study has reported an increase in the susceptibility of injury to the major neurovascular structures after partial or complete calviculectomy. In a study by Li et al., the functional outcomes and complication rates of 11 patients with clavicular tumors treated with partial or complete calviculectomy were studied. Out of the six patients who received clavicle reconstruction after excision, two developed nonunion and one developed an infection. The authors attributed these complications to avascular necrosis after the administration of high doses of chemotherapy after surgery [15]. Other possible complications to be considered include donor site morbidity, implant failure, and injury to the nearby neurovascular structures [16].

Multiple studies have investigated the clinical and functional outcomes after partial or complete claviculectomy without reconstruction. Most of these studies have reported good to excellent functional outcomes of the upper limb with absent to minimal postoperative pain and complications [1-3,5,9,10,12,14-19]. In a retrospective study on 44 patients with clavicular lesions by Korkmaz et al, 15 patients underwent claviculectomy [10]. The mean musculoskeletal tumor society (MSTS) score of these 15 patients was 79.4, indicating excellent functional outcomes. These results were similar to other studies reporting outcomes in the same range using the same outcome score [5,15]. Similarly, Smolle et al. reported the functional outcomes of 15 patients after partial or complete claviculectomy using the MSTS score and QuickDASH (Quick Disabilities of Arm, Shoulder & Hand) score. The mean MSTS and QuickDASH scores were 26 and 18 points on average, respectively, with slightly better scores in the partial group compared to the complete claviculectomy group [16].

Survival rates post-management of Ewing sarcoma depend on the presence of metastasis at the time of presentation, age at presentation, presence of positive margins after radical surgical excision, and response to chemotherapy [5,14,18]. In the study by Gulia et al. on 21 patients with Ewing sarcoma of the clavicle treated with chemotherapy and radical excision of the tumor with or without radiation, the five-year survival was 64% in patients with no metastasis as compared to 33% in patients with limited metastasis [5].

## Conclusions

Tumors of the clavicle are uncommon lesions with a histopathological profile quite different from that seen in other long or flat bones. A high index of suspicion is needed for the timely diagnosis and management of tumors at these rare sites since early detection and treatment before metastasis of the tumor will improve the chances of survival. Local control of the disease is achieved by the wide-margin radical resection of the tumor and the underlying bone by either partial or complete claviculectomy, to ensure tumor-free margins. Based on the currently available evidence, reconstruction of the clavicle with an allograft or a vascularized fibular graft is unnecessary after excision due to the high risks of complications and the excellent functional results of both partial and total claviculectomy alone.
